# Multiple Power Allocation Game Schemes for Spectrum Coexistence Model Between Multistatic MIMO Radar Sensors and MU Communication

**DOI:** 10.3390/s20216216

**Published:** 2020-10-31

**Authors:** Bin He, Hongtao Su

**Affiliations:** National Laboratory of Radar Signal Processing, Xidian University, Xi’an 710071, China; suht@xidian.edu.cn

**Keywords:** power allocation, spectrum coexistence, game theory, nash equilibrium, radar and communication

## Abstract

The normal operations of radar systems and communication systems under the condition of spectrum coexistence are facing a huge challenge. This paper uses game theory to study power allocation problems between multistatic multiple-input multiple-output (MIMO) radars and downlink communication. In the case of spectrum coexistence, radars, base station (BS) and multi-user (MU) have the working state of receiving and transmitting signals, which can cause unnecessary interferences to different systems. Therefore, when they work together, they should try to suppress mutual interferences. Firstly, the signal from BS is considered as interference when radar detects and tracks targets. A supermodular power allocation game (PAG) model is established and the existence and uniqueness of the Nash equilibrium (NE) in this game are proved. In addition, the power allocation problem from BS to MU is also analyzed, and two Stackelberg PAG models are constructed. It is proved that the NE of each game exists and is unique. Simultaneously, two Stackelberg power allocation iterative algorithms converge to the NEs. Finally, numerical results verify the convergence of the proposed PAG algorithms.

## 1. Introduction

Both radar systems and communication systems work with radio signals. Due to the lack of radio spectrum resources, radar and communication usually have to work under the same spectrum resources at the same time. In this case, the common spectrum between radar and communication will be crowded and interfered with each other, which affects their normal operation and reduces their work efficiency. In order to further improve the normal operation ability and reduce the mutual interferences between radar and communication, a reasonable power allocation strategy needs to be proposed by solving this problem [1,2,3,4,5].

The main research of modern radar communication integration includes radar embedded communication, integrated radar communication and radar communication coexistence, and the research contents include not only the cooperation, but also the mutual interferences between radar and communication. In order to improve the cooperation ability as much as possible and suppress the interferences between them, many scholars have done a lot of research work, mainly including waveform design [6,7,8,9,10,11,12,13,14], beamformer design [15,16,17,18,19], resource allocation [20,21,22], performance analysis of joint system [23,24,25,26,27,28,29,30,31] and so on. In [6], a multi-objective optimization problem is established by using the Cramer Rao bound (CRB). Furthermore, a method for joint adaptive weight optimization and Pareto optimization waveform design is proposed to improve the accuracy of radar range and speed estimation and the channel capacity of communication system. In [8], the waveform design of a dual function radar communication system for target detection and downlink communication is discussed, and two waveform design methods for minimizing multi-user (MU) interferences are proposed to solve the problem of constant mode waveform design in practice. In addition, communication waveform is embedded into the radar to construct a radar communication integrated system. A radar waveform design strategy with orthogonal frequency modulation embedded communication symbols is proposed. The experiment shows that it has reduced bit error rate (BER) [11]. In the beamformer design research, a novel multi-beam framework is established by using the guided analog antenna array. Simultaneously, the corresponding beamformer design and sensing algorithm is proposed to meet the requirements for joint communication and sensing system [15]. In [16], the hybrid beamformer design of dual function radar communication system in the mm band is studied. Given the optimal communication beamformer and the expected beampattern of radar, the authors propose to design an analog and digital beamformer by minimizing the weight sum of radar communication beamformer error under the condition of non-convex constant mode and power constraint. In addition, the authors of [17] consider a spectrum sharing case of joint multiple-input multiple-output (MIMO) radar and MU-MIMO communication. A robust beamforming optimization method is proposed to maximize the detection probability for a given performance requirement of downlink communication system. Next, for the problem of radar-communication resource allocation, a method for joint beam design of base station (BS) and power allocation is proposed, which maximizes the radar detection probability and guarantees the quality of service (QoS) and power budget for each user [20]. In [21], a Stackelberg game is used to study the power allocation problem of multistatic radar and communication system under spectrum coexistence. An iterative power allocation algorithm is proposed and converges to the Nash equilibrium (NE), which can minimize the radar transmit power by giving the desired signal to interference noise ratio (SINR) of a radar system and the interference limit of a communication system. Accordingly, the authors of [22] propose a wireless power integrated radar communication system for the energy limitation of integrated radar and communication system. An optimization problem for minimizing the total energy and guaranteeing the performance constraints of the integrated system is studied, and the optimal solution of the model is obtained by semi-definite relaxation of non-convex problem. Considering two spectrum sharing cases, the authors of [23] propose a target detection method based on zero space mapping null-space projection (NSP) waveform, which the method is compared with the target detection method for orthogonal waveform. The results show the superiority of the target detection ability of the NSP. On the basis of literature [23], Awais khawar et al. continue to analyze the coexistence of radar and cellular communication system under line of sight (LOS). They use the NSP method to reduce the impact of interference on the system [25]. In [24], the authors propose a new method to generate performance boundary of joint radar and communication system. The properties of several different inner boundaries are studied, including the SiC inner boundary, the isolated sub band inner boundary, the communication water-filling inner boundary and the optimal Fisher information inner boundary. Moreover, He et al. study the CRB and mutual information (MI) of cooperative MIMO radar and MIMO communication, and analyze the performance gain of the joint system [30].

In resource allocation, game theory is introduced into the fields of communication and radar as an effective resource allocation optimization theory [32,33,34,35,36,37,38]. In the field of communication, literature [32] studies the uplink power control of code division multiple access (CDMA) using the non-cooperative game theory, and proposes two algorithms to update strategies and converge to a stable NE point. In [33], an optimal downlink beamformer strategy for each BS is determined greedily by a distributed method without any cooperation, and a strategy non-cooperative game (SNG) is constructed. Then, the existence and uniqueness of the NE is proved by using the theoretical framework of standard function. With regard to the power control problem of time of arrival (TOA), the authors of [34] establish a supermodular game. They propose a distributed power allocation algorithm based on the supermodular game and prove that there is a unique NE solution. In addition, in the field of radar research, literature [35] uses game theory to analyze the interaction between radar and jamming. The authors use detection probability and false alarm probability to construct the utility function of the game and obtain supermodular and submodular games. There are pure strategic NE solutions for both games. In the presence of MIMO radar and multi-target, a distributed beamforming and power allocation technique is studied in [36]. Under a certain detection criterion, the authors establish non-cooperative game, partial non-cooperative game and Stackelberg game, and prove the existence and uniqueness of NE solutions of these games. The authors of [37] use cooperative game theory to study an optimal power allocation problem of distributed MIMO radar network under target tracking, and verify that the cooperative game power allocation method is superior to random power allocation and uniform power allocation.

In this paper, the power allocation problems between multistatic MIMO radar and MU communication are investigated in the case of spectrum coexistence. Due to the lack of radio spectrum resources, the two systems may interfere with each other. In order to reduce the interferences between the two system, we use game theory to propose different power allocation strategies. For clarification, we list the main work of this paper as follows:

- Three game frameworks of power allocation between a multistatic MIMO radar network and MU communication are built, including supermodular power allocation game (PAG), Stackelberg PAG 1 and Stackelberg PAG 2, which help decision makers to make different strategy choices under different power allocation situations.
- The utility functions of radar system and communication system are constructed, respectively. The closed-form solutions of the best response (BR) strategies of radar and communication are obtained by solving the utility function, respectively.
- Based on game theoretic analysis, the existence and uniqueness of the NEs of these games are strictly proved.
- Three PAG algorithms are proposed, which converge to the NEs of the games. Numerical results verify the effectiveness and convergence of these algorithms.

The rest of this paper is organized as follows. The system model is presented in Section 2. Section 3 constructs the PAG between radar system and communication system, and proves the existence and uniqueness of the NE. In addition, a supermodular PAG algorithm is proposed and converges to the NE. Similarly, Section 4 establishes two Stackelberg game models which the existence and uniqueness of the NE are also proved. Section 5 uses numerical results to verify the convergence of the proposed algorithms. Finally, Section 6 concludes this paper.

Notation: ${\left(•\right)}^{T}$, ${\left(•\right)}^{*}$ and ${\left(•\right)}^{H}$ represent the transpose, conjugate and conjugate transpose operation, respectively. ${∥•∥}_{F}$ defines the Frobenius norm. ∥•∥ defines the Euclidean norm. E denotes the mathematical expectation.

## 2. System Model

**Figure 1:**
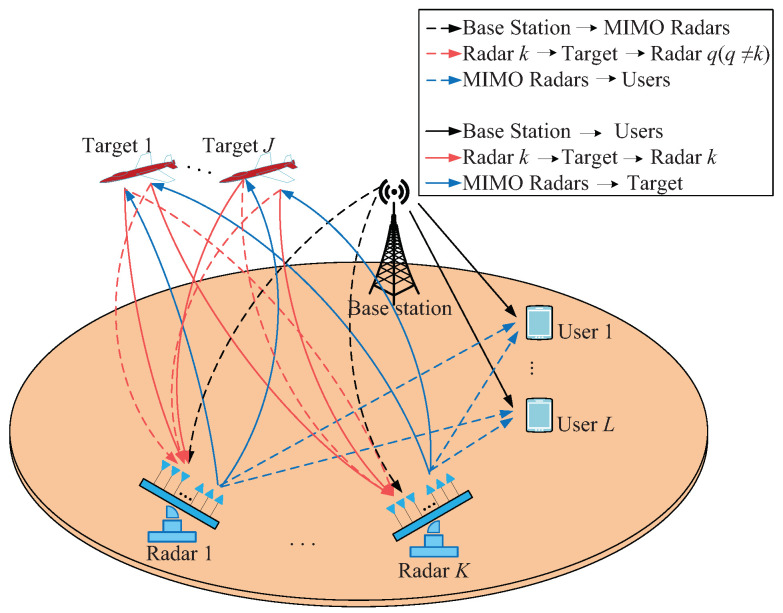
Multistatic multiple-input multiple-output (MIMO) radar network with multi-user (MU) communication.

As shown in Figure 1, this paper considers a spectrum coexistence model among multistatic MIMO radars, BS and MU. In this paper, each radar of the multistatic MIMO radar network has a uniform linear array of $M_{t}$ transmit antennas and $M_{r}$ receive antennas. In particular, it is assumed that each radar is equipped with the same number of transmit and receive antennas $M=M_{t}=M_{r}$, and the spacing between adjacent elements is half wavelength. By controlling the digital transceiver units of the MIMO radars, the waveforms transmitted by each element to different targets meet the orthogonality. Therefore, due to the orthogonality of the signal, it can not do the same phase superposition to synthesize the narrow beam with high gain in spatial domain, but can form the wide beam with low gain. Meanwhile, all array elements will form a digital multi-beam at the receiving end. In order to make full use of the transmitting energy, the multi-beam of the receiver can be used to cover the wide beam space of the transmitter. In addition, when the radar performs the task of tracking the target, it needs to transmit a certain amount of energy signal to capture the target. Therefore, the signal transmitted by the radar also interferes with the downlink MU receiver. On the communication side, we consider the downlink communication from BS to MU. The BS transmits useful signals to users, but it will interfere with the radar receiver. In order to reduce the mutual interference between radar system and communication system, both sides need to optimize resource allocation to better solve the interference problem. Furthermore, the system model is constructed to analyze the power allocation problem under spectrum coexistence.

Firstly, in a multistatic MIMO radar system, the precoding waveform vector of the *k*-th radar is $\psi_{Rk}\left(t\right)={\left[\sqrt{p_{R(k1)}}\psi_{R(k1)}\left(t\right),\cdots ,\sqrt{p_{R(kJ)}}\psi_{R(kJ)}\left(t\right)\right]}^{T}$, where $p_{R(kj)}$ is the signal power transmitted by the *k*-th radar to the *j*-th target. *t* is time index of radar pulse. In addition, the waveform satisfies the orthogonality $\int_{T_{R}}\psi_{R(kj)}\left(t\right)\psi_{R(kj)}^{*}\left(t\right)dt=1$, where $T_{R}$ denotes the pulse width of the radar. To satisfy the orthogonality conditions of the waveforms, the array of the MIMO radar needs to provide more degrees of freedom (DoF) than the phased array radar. Therefore, it is necessary to transmit many streams from the MIMO radar to different targets. Furthermore, the waveform vector transmitted by the *k*-th MIMO radar to the *j*-th target is obtained as follows:

(1) $$s_{R(kj)}\left(t\right)=w_{t(kj)}\psi_{R(kj)}\left(t\right)$$

where $w_{t(kj)}$ is the normalized transmit beamformer weight vector from the *k*-th radar to the *j*-th target. The *k*-th MIMO radar receiving signal corresponding to the *j*-th target can be obtained as follows:

(2) $$\begin{array}{c} y_{R(kj)}\left(t\right)=\sum_{q=1}^{K}\sum_{i=1}^{J}\sqrt{p_{R(qi)}}w_{r(kj)}^{H}A_{{}_{kqi}}s_{R(qi)}\left(t\right)+\sum_{l=1}^{L}\sqrt{p_{Bl}}w_{r(kj)}^{H}A_{Bk}s_{Bl}\left(t\right)+n_{R(kj)}\left(t\right) \end{array}$$

where $p_{Bl}$ represents the transmitted signal power from the communication BS to the *l*-th user, $n_{R\left(kj\right)}$ denotes additive white Gaussian noise, $n_{R(kj)}∼\mathrm{CN}\left(0,\sigma_{R}^{2}\right)$.

In addition, the precoding vector of the BS to the users is $\psi_{B}\left(t\right)={\left[\sqrt{p_{B1}}\psi_{B1}\left(t\right),\cdots ,\sqrt{p_{BL}}\psi_{BL}\left(t\right)\right]}^{T}$. $\psi_{Bl}\left(t\right)$ is the communication symbol which satisfies the orthogonality $E\left\{\psi_{Bl}\psi_{Bl}^{*}\right\}=1$. Furthermore, the symbol transmitted by the BS to the *l*-th user is obtained as follows:

(3) $$\begin{array}{c} s_{Bl}\left(t\right)=w_{Bl}\psi_{Bl}\left(t\right) \end{array}$$

where $w_{Bl}$ is the normalized transmit beamformer weight vector of the BS corresponding to the *l*-th user. The correlation between radar waveform and communication symbol has the relationship of $\int_{T_{R}}\psi_{R(kj)}\left(t\right)\psi_{Bl}^{*}\left(t\right)dt=\epsilon ,\forall (kj),l$, where ϵ is a relatively small number. In addition, the transmit–receive vector matrix A is expressed as:

(4) $$\begin{array}{c} A_{kqi}=a_{r}\left(\theta_{ki}\right)a_{t}^{H}\left(\theta_{qi}\right)\beta_{i} \end{array}$$

(5) $$\begin{array}{c} A_{Bk}=a_{r}\left(\theta_{Rk}\right)h_{Bk}^{H}\left(\theta_{Bk}\right) \end{array}$$

where $\beta_{i}$ is the scatter amplitude of the *i*-th target, $\theta_{ki}$ denotes the azimuthal angle of the *i*-th target by considering the *k*-th radar as reference, $\theta_{Bk}$ is the angle of arrival (AoA) from the *k*-th radar to the BS, $\theta_{Rk}$ is the AoA from the BS to the *k*-th radar, $a_{r}\left(\theta \right)$ and $a_{t}\left(\theta \right)$ are the receive and transmit steering vectors, respectively, which are expressed as follows:

$$\begin{array}{c} a_{r}\left(\theta \right)={\left[1\;e^{jd\frac{2\pi }{\lambda }\sin\theta }\;\cdots \;e^{j\left(M_{r}-1\right)d\frac{2\pi }{\lambda }\sin\theta }\right]}^{T}\\ a_{t}\left(\theta \right)={\left[1\;e^{jd\frac{2\pi }{\lambda }\sin\theta }\;\cdots \;e^{j\left(M_{t}-1\right)d\frac{2\pi }{\lambda }\sin\theta }\right]}^{T} \end{array}$$

where *d* is the distance between adjacent elements, λ is the wavelength of the transmitted signal, θ describes the AoA. $h_{Bk}$ is the jamming channel vector from the communication BS to the *k*-th radar, which is defined as follows:

$$h_{Bk}={\left[1\;e^{jd\frac{2\pi }{\lambda }\sin\theta_{Bk}}\;\cdots \;e^{j\left(M-1\right)d\frac{2\pi }{\lambda }\sin\theta_{Bk}}\right]}^{T}$$

Furthermore, the receive SINR of the *k*-th radar to the *j*-th target can be expressed as:

(6) $$\gamma_{R(kj)}=\frac{p_{R(kj)}{∥u_{kkj}∥}^{2}}{p_{B}{∥u_{Bk}∥}^{2}+\eta_{R}}$$

where $\eta_{R}=\sum_{q\neq k}^{K}\sum_{i=1}^{J}p_{R(qi)}∥u_{kqi}{∥}^{2}+\sum_{i\neq j}^{J}p_{R(ki)}{∥u_{kki}∥}^{2}+{\tilde{\sigma }}_{R}^{2}$, ${\tilde{\sigma }}_{R}^{2}=\sum_{q=1}^{K}\sum_{i=1}^{J}\sum_{l=1}^{L}\sqrt{p_{Bl}}\sqrt{p_{R(qi)}}\left|u_{kqi}^{H}u_{kki}\right|\epsilon +\sigma_{R}^{2}$, $u_{kqi}=w_{r(kj)}^{H}A_{{}_{kqi}}$, $u_{Bk}=w_{r(kj)}^{H}A_{Bk}$, $\sum_{l=1}^{L}p_{Bl}=p_{B}$. $\sigma_{R}^{2}$ is the noise variance of the *k*-th radar. The total interferences except being transmitted by the *k*-th radar to the *j*-th target and received by the *k*-th radar can be expressed as:

(7) $$I_{-R(kj)}=p_{B}{∥u_{Bk}∥}^{2}+\eta_{R}$$

Then, according to Equations (6) and (7), there are the following results:

(8) $$\begin{array}{c} \gamma_{R(kj)}=\frac{p_{R(kj)}{∥u_{kkj}∥}^{2}}{I_{-R(kj)}} \end{array}$$

(9) $$\begin{array}{c} \frac{\partial \gamma_{R(kj)}}{\partial p_{R(kj)}}=\frac{∥u_{kkj}{∥}^{2}}{I_{-R(kj)}}=\frac{\gamma_{R(kj)}}{p_{R(kj)}} \end{array}$$

(10) $$\begin{array}{c} \frac{\partial \gamma_{R(kj)}}{\partial p_{B}}=-\frac{p_{R(kj)}{∥u_{kkj}∥}^{2}∥u_{Bk}{∥}^{2}}{I_{-R(kj)}^{2}} \end{array}$$

On the other hand, this paper considers the case of downlink MU in the communication system. Furthermore, the MU will receive the useful signals from the BS and the jamming signals from the radars. Therefore, the receiving signal model of the *l*-th user is:

(11) $$\begin{array}{c} y_{l}\left(t\right)=\sum_{l=1}^{L}\sqrt{p_{Bl}}h_{Cl}^{H}s_{Bl}\left(t\right)+\sum_{k=1}^{K}\sum_{j=1}^{J}\sqrt{p_{R(kj)}}a_{R(kl)}^{H}s_{R(kj)}\left(t\right)+n_{l}\left(t\right) \end{array}$$

where $n_{l}∼\mathrm{CN}\left(0,\sigma_{U}^{2}\right)$ represents the noise of the *l*-th user’s receiver, $h_{Cl}$ denotes the channel vector from the BS to the *l*-th user and can be expressed as follows:

$$h_{Cl}={\left[1\;e^{jd\frac{2\pi }{\lambda }\sin\theta_{Cl}}\;\cdots \;e^{j\left(M-1\right)d\frac{2\pi }{\lambda }\sin\theta_{Cl}}\right]}^{T}$$

where $\theta_{Cl}$ denotes the AoA from the *l*-th user to the BS, and $u_{R(kl)}$ denotes the jamming channel vector from the *k*-th radar to the *l*-th user and can be defined as follows:

$$a_{R(kl)}={\left[1\;e^{jd\frac{2\pi }{\lambda }\sin\theta_{R(kl)}}\;\cdots \;e^{j\left(M_{t}-1\right)d\frac{2\pi }{\lambda }\sin\theta_{R(kl)}}\right]}^{T}$$

where $\theta_{R(kl)}$ denotes the azimuthal angle of the *l*-th user by considering the *k*-th radar as reference. Furthermore, the receive SINR of the *l*-th downlink user is:

(12) $$\gamma_{Cl}=\frac{Gp_{Bl}{∥h_{Cl}∥}^{2}}{\sum_{m\neq l}^{L}p_{Bm}{∥h_{Cm}∥}^{2}+\eta_{U}}$$

where $\eta_{U}=\sum_{k=1}^{K}\sum_{j=1}^{J}p_{R(kj)}{∥a_{R(kl)}∥}^{2}+{\tilde{\sigma }}_{U}^{2}$, ${\tilde{\sigma }}_{U}^{2}=\sum_{k=1}^{K}\sum_{j=1}^{J}\sum_{l=1}^{L}\sqrt{p_{Bl}}\sqrt{p_{R(kj)}}\left|h_{Cl}^{H}a_{R(kl)}\right|\epsilon +\sigma_{U}^{2}$. $\sigma_{U}^{2}$ is the noise variance of the *l*-th user. *G* is spread spectrum gain. The total interferences received by the *l*-th user can be written as follows:

(13) $$I_{-Cl}=\sum_{m\neq l}^{L}p_{Bm}{∥h_{Cm}∥}^{2}+\eta_{U}$$

In the next section, the game theory framework is established. We will use game theory to analyze the power allocation problem between radar system and communication system, and prove the existence and uniqueness of the NE.

## 3. Supermodular Power Allocation Game

A power allocation game theory framework is constructed in this section. First of all, in order to solve the problem of radar jammed by communication BS, we establish a supermodular PAG between the radar and the BS. The players of the game are the multistatic MIMO radar system and the BS. The strategies of the game are the signal power transmitted by both sides. The supermodular game is constructed as follows:

(14) $$G_{1}=\left\{P_{1},S_{1},U_{1}\right\}$$

1. Player: $P_{1}=\left\{Radar,Communication\right\}$.
2. Strategy: $S_{1}=S_{R}\times S_{B}$, $S_{R}=\left\{p_{R}\right\}$, $S_{B}=\left\{p_{B}\right\}$, $p_{R}={\left[p_{R1},\cdots ,p_{RK}\right]}^{T}$, $p_{Rk}={\left[p_{R(k1)},\cdots ,p_{R(kJ)}\right]}^{T}$, $p_{B}={\left[p_{B1},\cdots ,p_{BL}\right]}^{T}$.
3. Utility function: $U_{1}=\left\{U_{R(kj)}\right\}$.

where $p_{Rk}$ is the transmit power vector of the *k*-th radar, $p_{B}$ is the transmit power vector of the BS

Then, the utility function of the *k*-th radar corresponding to the *j*-th target is defined as a log function to meet the target detection performance as follows:

(15) $$\begin{array}{c} U_{R(kj)}\left(p_{R(kj)},p_{B}\right)=\ln\left(\gamma_{R(kj)}-\gamma_{R(kj)}^{\min}\right)-\varepsilon_{kj}p_{R(kj)}-\omega_{k}p_{B} \end{array}$$

where $\gamma_{R(kj)}^{\min}$ is the minimum SINR threshold, $\varepsilon_{kj}$ and $\omega_{k}$ are the linear price factor coefficients which are positive.

Therefore, the game optimization model is constructed by maximizing the utility function as:

(16) $$\begin{array}{cc} \max_{p_{Rk}}\min_{p_{B}} & \sum_{j=1}^{J}U_{R(kj)}\left(p_{R(kj)},p_{B}\right)\\ s.t.\; & \gamma_{R(kj)}\geq \gamma_{R(kj)}^{\min},\;\forall j\\ \; & 0\leq p_{R(kj)}\leq p_{R(kj)}^{\max},\;\forall j\\ \; & 1_{J}^{T}p_{Rk}\leq p_{Rk}^{\mathrm{Tot}}\\ \; & 0\leq p_{B}\leq p_{B}^{\max} \end{array}$$

where $p_{R(kj)}^{\max}$ is the maximum transmitting power of the *k*-th radar to the *j*-th target, $p_{Rk}^{\mathrm{Tot}}$ is the total transmit power of the *k*-th radar, $1_{J}$ is all one vector whose length is *J*, $p_{B}^{\max}$ denotes the maximum transmitting power from the BS.

According to the game optimization model, it is necessary to obtain an iterative power allocation equation of radar. The best response function of the radar is written as:

(17) $$\begin{array}{c} p_{R(kj)}^{*}=\arg\max_{p_{R(kj)}}U_{R(kj)}\left(p_{R(kj)},p_{B}\right) \end{array}$$

Therefore, the best power response strategy of the *k*-th radar corresponding to the *j*-th target is as follows:

(18) $$p_{R(kj)}^{*}=\frac{\gamma_{R(kj)}^{\min}I_{-R(kj)}}{∥u_{kkj}{∥}^{2}}+\frac{1}{\varepsilon_{kj}}$$

Similarly, for the BS power allocation strategy, the best response function is obtained as:

(19) $$\begin{array}{c} p_{B}^{*}=\arg\min_{p_{B}}U_{R(kj)}\left(p_{R(kj)},p_{B}\right) \end{array}$$

Therefore, according to Equation (19), the quadratic equation of one unknown variable is obtained as follows:

(20) $$\begin{array}{c} I_{-R(kj)}^{2}-\frac{p_{R(kj)}{∥u_{kkj}∥}^{2}}{\gamma_{R(kj)}^{\min}}I_{-R(kj)}-\frac{p_{R(kj)}{∥u_{kkj}∥}^{2}{∥u_{Bk}∥}^{2}}{\omega_{k}\gamma_{R(kj)}^{\min}}=0 \end{array}$$

According to the root formula of quadratic equation of one variable, it is $x_{1,2}=\frac{1}{2a}\left(-b\pm \sqrt{b^{2}-4ac}\right)$. In order to ensure that the power is positive, the plus sign solution is taken as the following equation:

(21) $$\begin{array}{c} I_{-R(kj)}^{*}=\frac{p_{R(kj)}{∥u_{kkj}∥}^{2}}{2\gamma_{R(kj)}^{\min}}+\sqrt{{\left(\frac{p_{R(kj)}{∥u_{kkj}∥}^{2}}{2\gamma_{R(kj)}^{\min}}\right)}^{2}+\frac{p_{R(kj)}{∥u_{kkj}∥}^{2}{∥u_{Bk}∥}^{2}}{\omega_{k}\gamma_{R(kj)}^{\min}}} \end{array}$$

Combined with Equation (7), the power allocation equation of the BS is obtained as follows:

(22) $$p_{B}^{*}=\frac{1}{{∥u_{Bk}∥}^{2}}\left(I_{-R(kj)}^{*}-\eta_{R}\right)$$

**Figure 2:**
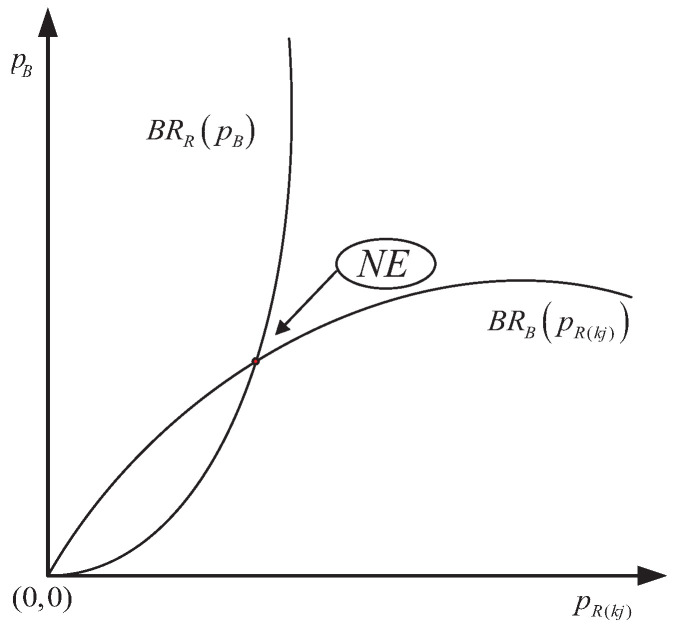
Nash equilibrium for supermodular power allocation game (PAG).

Therefore, according to the game theory, the best power allocation strategy solutions of the radar and the BS are obtained, and the NE solution is shown as Figure 2.

For the supermodular game, the utility function of the game needs to satisfy a increasing difference relationship for the game strategies, which is equivalent to the second-order mixed partial derivative of the utility function with respect to both strategies being greater than zero [34]. Therefore, the second-order mixed partial derivative of the utility function with respect to the radar power and the BS power is calculated as follows:

(23) $$\begin{array}{cc} \frac{\partial^{2}U_{R(kj)}\left(p_{R(kj)},p_{B}\right)}{\partial p_{B}\partial p_{R(kj)}}\; & =\frac{\partial \left(\frac{\gamma_{R(kj)}}{p_{R(kj)}\left(\gamma_{R(kj)}-\gamma_{R(kj)}^{\min}\right)}-\varepsilon_{kj}\right)}{\partial p_{B}}\\ \; & =-\frac{\frac{\partial \gamma_{R(kj)}}{\partial p_{B}}\gamma_{R(kj)}^{\min}}{p_{R(kj)}{\left(\gamma_{R(kj)}-\gamma_{R(kj)}^{\min}\right)}^{2}}\\ \; & =\frac{∥u_{kkj}{∥}^{2}{∥u_{Bk}∥}^{2}\gamma_{R(kj)}^{\min}}{{\left(\gamma_{R(kj)}-\gamma_{R(kj)}^{\min}\right)}^{2}I_{-R(kj)}^{2}}\geq 0 \end{array}$$

Furthermore, the game belongs to the supermodular game, which has the pure strategy NE, so the existence of the NE is satisfied. Next, the uniqueness of the NE is proved by the definition of standard function. The standard function satisfies the following three properties:

1. Positivity: The function is strictly positive, F(x)>0.
2. Monotonicity: If $x^{\prime }\geq x$, then $F\left(x^{\prime }\right)\geq F\left(x\right)$.
3. Scalability: For all a>1, it has aF(x)>F(ax).

The best power allocation strategy of the *k*-th radar is derived as Equation (24).

(24) $$\begin{array}{c} BR_{R}\left(p_{B}\right)=\frac{\gamma_{R(kj)}^{\min}}{∥u_{kkj}{∥}^{2}}\left(p_{B}{∥u_{Bk}∥}^{2}+\eta_{R}\right)+\frac{1}{\varepsilon_{\left(kj\right)}} \end{array}$$

**Proof.**  First, the best power response function of the radar system communication system holds the following properties for all $p_{R}>0$ and $p_{B}>0$:

(a) Positivity: For any $p_{R(kj)}>0$ and $p_{B}>0$, then $BR_{R}\left(p_{B}\right)>0$.
(b) Monotonicity: If $p_{B}^{\prime }\geq p_{B}$, then
  $$\begin{array}{c} BR_{R}\left(p_{B}^{\prime }\right)-BR_{R}\left(p_{B}\right)=\frac{\gamma_{R(kj)}^{\min}{∥u_{Bk}∥}^{2}}{∥u_{kkj}{∥}^{2}}\left(p_{B}^{\prime }-p_{B}\right)\geq 0 \end{array}$$
(c) Scalability: For all a>1, it has
  $$aBR_{R}\left(p_{B}\right)-BR_{R}\left(ap_{B}\right)=\left(a-1\right)\left(\frac{\eta_{R}\gamma_{Rk}^{\min}}{∥u_{kkj}{∥}^{2}}+\frac{1}{\varepsilon_{kj}}\right)>0$$
   □

Thus, the existence and uniqueness of the NE of the game are proved. After many iterations of the game, the optimal power allocation strategies of both sides converge to a fixed NE solution. Furthermore, the equation for the *k*-th radar power allocation can be written as:

(25) $$\begin{array}{c} p_{R(kj)}^{\left(n+1\right)}={\left[\frac{\gamma_{R(kj)}^{\min}}{∥u_{kkj}{∥}^{2}}\left(p_{B}^{\left(n\right)}{∥u_{Bk}∥}^{2}+\eta_{R}^{\left(n\right)}\right)+\frac{1}{\varepsilon_{\left(kj\right)}}\right]}_{0}^{p_{R(kj)}^{\max}} \end{array}$$

where *n* is the step of iteration, $\eta_{R}^{\left(n\right)}=\sum_{q\neq k}^{K}\sum_{i=1}^{J}p_{R(qi)}^{\left(n\right)}∥u_{kqi}{∥}^{2}+\sum_{i\neq j}^{J}p_{R(ki)}^{\left(n\right)}{∥u_{kki}∥}^{2}+{\tilde{\sigma }}_{R}^{2}$, and ${\left[x\right]}_{a}^{b}=\max\left[\min\left[x,b\right],a\right]$. Then, the iterative formula of the radar power allocation can be obtained by using variable substitution as follows:

(26) $$p_{R(kj)}^{\left(n+1\right)}={\left[\frac{\gamma_{R(kj)}^{\min}}{\gamma_{R(kj)}}p_{R(kj)}^{\left(n\right)}+\frac{1}{\varepsilon_{\left(kj\right)}}\right]}_{0}^{p_{R(kj)}^{\max}}$$

In addition, according to Equation (22), the corresponding BS power allocation formula is as follows:

(27) $$p_{B}^{\left(n+1\right)}=\frac{1}{{∥u_{Bk}∥}^{2}}{\left[I_{-R(kj)}^{*(n+1)}-\eta_{R}^{\left(n\right)}\right]}_{0}^{p_{B}^{\max}}$$

Based on the above research and analysis, an iterative supermodular PAG algorithm is proposed, which converges to the NE of the game. The algorithm is based on two level iterations. The transmit power of the multistatic MIMO radar network is a non-cooperative game with inner layer. Additionally, the outer layer is the power allocation of the BS corresponding to the radar. Therefore, the iterative algorithm is summarized in Algorithm 1.
**Algorithm 1** Supermodular PAG Algorithm. 1: **Input** Set the parameters and initial power 2: **repeat** 3:  **for**
k←1,K
**do** 4:    **repeat** 5:      **for**
j←1,J,k←1,K
**do** 6:       Calculate $p_{R(kj)}^{\left(n\right)}$, based on Equation (26) 7:      **end for** 8:    **until**
$\left|p_{R(kj)}^{\left(n+1\right)}-p_{R(kj)}^{\left(n\right)}\right|<\delta$ 9:    Calculate $p_{B}^{\left(n\right)}$, based on Equation (27) 10:   **end for** 11: **until**$\left|p_{B}^{\left(n+1\right)}-p_{B}^{\left(n\right)}\right|<\delta$ 12: Output
$p_{R(kj)}$, $p_{B}$.


In conclusion, this section establishes a framework of supermodular PAG and constructs the utility function of the game. Then, a power allocation constraint optimization model is established, and the pure strategy NE of the game is analyzed by using supermodular game theory. The uniqueness of the NE of the game is also proved. Finally, a supermodular PAG algorithm is proposed.

## 4. Stackelberg Power Allocation Game

### 4.1. Stackelberg PAG Algorithm 1

In this subsection, the problem that the downlink MU is interfered by the radar transmit signals. We also construct the utility function of MU, and establish a MU power allocation optimization model. Then, two Stackelberg game algorithms are established. The first algorithm is that the multistatic MIMO radar is the leader of the game, and the BS is served as the follower to allocate power to different downlink users. Reversely, the second algorithm is the communication system as the leader and the radar as the follower. Next, the first Stackelberg game theory framework is constructed as follows:

(28) $$G_{2}=\left\{P_{2},S_{2},U_{2}\right\}$$

1. Player: $P_{2}=\left\{Radar,Communication\right\}$.
2. Strategy: $S_{2}=S_{R}\times S_{C}$, $S_{R}=\left\{p_{R}\right\}$, $S_{C}=\left\{p_{B}\right\}$, $p_{R}={\left[p_{R1},\cdots ,p_{RK}\right]}^{T}$, $p_{Rk}={\left[p_{R(k1)},\cdots ,p_{R(kJ)}\right]}^{T}$, $p_{B}={\left[p_{B1},\cdots ,p_{BL}\right]}^{T}$.
3. Utility function: $U_{2}=\left\{U_{R(kj)},U_{Cl}\right\}$.

A single cell system with *L* users is studied in this paper. The number of users is limited under an admission control strategy that ensures the minimum SINR for each user in the cell. Therefore, a logarithmic function is considered as the utility function of the *l*-th user:

(29) $$U_{Cl}\left(p_{Bl},p_{R(kj)}\right)=\mu_{l}\ln\left(1+\gamma_{Cl}\right)-\lambda_{l}{∥h_{Cl}∥}^{2}p_{Bl}$$

where $\mu_{l}$ denotes the *l*-th user level of “desire” for SINR, $\lambda_{l}$ is the linear pricing factor of the *l*-th user.

For the problem of MU power allocation optimization, the communication system expects to maximize its utility and meet a certain SINR threshold and power constraints. Therefore, we can construct the following optimization model:

(30) $$\begin{array}{cc} \max_{p_{B}}\min_{p_{R}} & \sum_{l=1}^{L}U_{Cl}\left(p_{Bl},p_{R(kj)}\right)\\ s.t.\; & \gamma_{Cl}\left(p_{Bl},p_{R(kj)}\right)\geq \gamma_{Cl}^{\min},\;\forall l\\ \; & 0\leq p_{Bl}\leq p_{Bl}^{\max},\;\forall l\\ \; & 1_{L}^{T}p_{B}\leq p_{B}^{\mathrm{Tot}}, \end{array}$$

where $1_{L}$ is all one vector whose length is *L*.

Next, in order to get the power update formula of the communication system, the first-order partial derivative of the utility function $U_{Cl}$ for the *l*-th user is obtained with respect to $p_{Cl}$, and makes it zero.

(31) $$\begin{array}{cc} \frac{\partial U_{Cl}\left(p_{Bl},p_{R(kj)}\right)}{\partial p_{Bl}}\; & =\mu_{l}\frac{\frac{\partial \gamma_{Cl}}{\partial p_{Bl}}}{1+\gamma_{Cl}}-\lambda_{l}{∥h_{Cl}∥}^{2}\\ \; & =\mu_{l}\frac{\gamma_{Cl}}{\left(1+\gamma_{Cl}\right)p_{Bl}}-\lambda_{l}{∥h_{Cl}∥}^{2}=0 \end{array}$$

If the SINR Equation (12) of user-*l* is further brought into the above Equation (31), then the following equation can be derived as:

(32) $$\frac{\mu_{l}G∥h_{Cl}{∥}^{2}}{Gp_{Bl}{∥h_{Cl}∥}^{2}+\sum_{m\neq l}^{L}p_{Bm}{∥h_{Cm}∥}^{2}+\eta_{U}}=\lambda_{l}{∥h_{Cl}∥}^{2}$$

Then, the above formula is sorted out to get the following power allocation equation:

(33) $$p_{Bl}{∥h_{Cl}∥}^{2}+\frac{\sum_{m\neq l}^{L}p_{Bm}{∥h_{Cm}∥}^{2}}{G}=\frac{\mu_{l}}{\lambda_{l}}-\frac{\eta_{U}}{G}$$

The above Equation (33) can be reformulated into the matrix form as follows:

(34) $$\begin{array}{c} \left[\begin{array}{cccc} 1 & \frac{1}{G} & \cdots & \frac{1}{G}\\ \frac{1}{G} & 1 & \cdots & \frac{1}{G}\\ \vdots & \vdots & \ddots & \vdots \\ \frac{1}{G} & \frac{1}{G} & \cdots & 1 \end{array}\right]\left[\begin{array}{c} p_{B1}{∥h_{C1}∥}^{2}\\ p_{B2}{∥h_{C2}∥}^{2}\\ \vdots \\ p_{BL}{∥h_{CL}∥}^{2} \end{array}\right]=\left[\begin{array}{c} \phi_{1}\\ \phi_{2}\\ \vdots \\ \phi_{L} \end{array}\right] \end{array}$$

where $\phi_{l}=\frac{\mu_{l}}{\lambda_{l}}-\frac{\eta_{U}}{G}$.

After the matrix form (34) is transformed, a new power allocation matrix form can be obtained as (35).

(35) $$\begin{array}{c} \left[\begin{array}{cccc} 1 & \frac{∥h_{C2}{∥}^{2}}{G∥h_{C1}{∥}^{2}} & \cdots & \frac{∥h_{CL}{∥}^{2}}{G∥h_{C1}{∥}^{2}}\\ \frac{∥h_{C1}{∥}^{2}}{G∥h_{C2}{∥}^{2}} & 1 & \cdots & \frac{∥h_{CL}{∥}^{2}}{G∥h_{C2}{∥}^{2}}\\ \vdots & \vdots & \ddots & \vdots \\ \frac{∥h_{C1}{∥}^{2}}{G∥h_{CL}{∥}^{2}} & \frac{∥h_{C2}{∥}^{2}}{G∥h_{CL}{∥}^{2}} & \cdots & 1 \end{array}\right]\left[\begin{array}{c} p_{B1}\\ p_{B2}\\ \vdots \\ p_{BL} \end{array}\right]=\left[\begin{array}{c} \frac{\phi_{1}}{∥h_{C1}{∥}^{2}}\\ \frac{\phi_{2}}{∥h_{C2}{∥}^{2}}\\ \vdots \\ \frac{\phi_{L}}{∥h_{CL}{∥}^{2}} \end{array}\right] \end{array}$$

Furthermore, let ${\left[G\right]}_{ll}=1$, ${\left[G\right]}_{lm}=\frac{∥h_{Cm}{∥}^{2}}{G∥h_{Cl}{∥}^{2}}$, for m≠l. $\Phi ={\left[\frac{\phi_{1}}{∥h_{C1}{∥}^{2}},\cdots ,\frac{\phi_{L}}{∥h_{CL}{∥}^{2}}\right]}^{T}$. According to [32], G is a nonsingular matrix, then G is invertible. Thus, the power allocation equation is as follows:

(36) $$p_{B}^{*}=G^{-1}\Phi$$

Based on the above discussion, we can get the power allocation from the BS to the *l*-th user as follows:

(37) $$p_{Bl}^{\left(n+1\right)}=\frac{1}{∥h_{Cl}{∥}^{2}}{\left[\phi_{l}^{\left(n+1\right)}-\frac{1}{G}\sum_{m\neq l}^{L}p_{Bm}^{\left(n\right)}{∥h_{Cm}∥}^{2}\right]}_{0}^{p_{Bl}^{\max}}$$

The best power response equation of the *l*-th user depends on the setting of user specific parameters, which is not only related to channel parameters, but also to network and price parameters. It is useful information for the signals transmitted by the BS to the MU, and the jamming signals transmitted by the radar to the MU. If the energy provided by the BS is relatively appropriate, the QoS of MU will be improved. According to the above analysis, the power allocation of MU is carried out by the radar system as a leader and the BS as a follower. Furthermore, an iterative Stackelberg game algorithm is proposed and converges to the NE. The algorithm is summarized as Algorithm 2.
**Algorithm 2** Stackelberg PAG Algorithm 1. 1: **Input** Set the parameters and initial power 2: **repeat** 3:  **for**
l←1,L
**do** 4:    **repeat** 5:      **for**
j←1,J, k←1,K
**do** 6:       Calculate $p_{R(kj)}^{\left(n\right)}$, based on Equation (26) 7:      **end for** 8:    **until**
$\left|p_{R(kj)}^{\left(n+1\right)}-p_{R(kj)}^{\left(n\right)}\right|<\delta$ 9:    Calculate $p_{Cl}^{\left(n\right)}$, based on Equation (37) 10:   **end for** 11: **until**$\left|p_{Bl}^{\left(n+1\right)}-p_{Bl}^{\left(n\right)}\right|<\delta$ 12: **Output**
$p_{R(kj)}$, $p_{Bl}$.


### 4.2. Stackelberg PAG Algorithm 2

In this subsection, another Stackelberg PAG is constructed. The BS is a leader and the radar system is a follower. Therefore, the Stackelberg game framework is constructed as follows:

(38) $$G_{3}=\left\{P_{3},S_{3},U_{3}\right\}$$

1. Player: $P_{3}=\left\{Communication,Radar\right\}$.
2. Strategy: $S_{3}=S_{C}\times S_{R}$, $S_{C}=\left\{p_{B}\right\}$, $S_{R}=\left\{p_{R}\right\}$, $p_{B}={\left[p_{B1},\cdots ,p_{BL}\right]}^{T}$$p_{R}={\left[p_{R1},\cdots ,p_{RK}\right]}^{T}$, $p_{Rk}={\left[p_{R(k1)},\cdots ,p_{R(kJ)}\right]}^{T}$.
3. Utility function: $U_{3}=\left\{U_{Cl},U_{Rk}\right\}$.

Similarly, for the Stackelberg PAG Algorithm 2, it also needs to satisfy the existence and uniqueness of the NE. On the basis of Equation (30), the second partial derivative of the utility function $U_{Cl}$ with respect to $p_{Cl}$ is obtained as follows:

(39) $$\begin{array}{cc} \frac{\partial^{2}U_{Cl}\left(p_{Bl},p_{R(kj)}\right)}{\partial p_{Bl}^{2}}\; & =\frac{\partial \left(\mu_{l}\frac{G∥h_{Cl}{∥}^{2}}{\left(1+\gamma_{Cl}\right)I_{-Cl}}-\lambda_{l}∥h_{Cl}{∥}^{2}\right)}{\partial p_{Bl}}\\ \; & =-\frac{\mu_{l}\gamma_{Cl}^{2}}{{\left(1+\gamma_{Cl}\right)}^{2}p_{Bl}^{2}}<0 \end{array}$$

Therefore, there is inner solution, if it exists, and is the unique point to minimize the utility function. In addition, the boundary solution is $p_{Bl}=0$ or $p_{Bl}=p_{Bl}^{\max}$, which is the other possible optimal solution for the constrained optimization problem (30). If the utility function obtains the maximum value, and its corresponding solution is $p_{Bl}^{*}<0$ or $p_{Bl}>p_{Bl}^{\max}$, its optimal solution will be the boundary solution [32]. Therefore, an iterative equation of power allocation from BS to the *l*-th user is obtained:

(40) $$\begin{array}{c} p_{{}_{Bl}}^{\left(n+1\right)}=\frac{1}{∥h_{Cl}{∥}^{2}}{\left[\phi_{l}^{\left(n\right)}-\frac{1}{G}\sum_{m\neq l}^{L}p_{Bm}^{\left(n\right)}∥h_{Cm}{∥}^{2}\right]}_{0}^{p_{Bl}^{\max}} \end{array}$$

According to the theorem of [32], the NE of the Stackelberg game exists and is unique. Furthermore, a parallel power update algorithm is constructed. The iteration formula of the algorithm is Equation (40), which converges to the NE solution of the game. Finally, the Stackelberg PAG Algorithm 2 is summarized as Algorithm 3.
**Algorithm 3** Stackelberg PAG Algorithm 2. 1: **Input** Set the parameters and initial power 2: **repeat** 3:  **for**
j←1,J,k←1,K
**do** 4:    **repeat** 5:      **for**
l←1,L
**do** 6:       Calculate $p_{Bl}^{\left(n\right)}$, based on Equation (40) 7:      **end for** 8:    **until**
$\left|p_{Bl}^{\left(n+1\right)}-p_{Bl}^{\left(n\right)}\right|<\delta$ 9:   Calculate $p_{R(kj)}^{\left(n\right)}$, based on Equation (25) 10:   **end for** 11: **until**$\left|p_{R(kj)}^{\left(n+1\right)}-p_{R(kj)}^{\left(n\right)}\right|<\delta$ 12: **Output**
$p_{R(kj)}$, $p_{Bl}$


In this section, we study the Stackelberg PAG between the radar and the communication. First, two Stackelberg game models are established. One is the game in which the radar system is the leader and the BS is the follower to allocate power to MU. Another is the game in which the BS is the leader and the radar system is the follower to allocate power to MU. The existence and uniqueness of the NEs of the two games are proved. Two kinds of Stackelberg PAG algorithms are proposed and converge to the NE solutions of the game.

## 5. Numerical Results

In this section, numerical results are presented to verify the convergence for the three PAG algorithms. It is assumed that each radar has the same number of transmit and receive antennas, and the distance between adjacent elements is half wavelength in the multistatic MIMO radar system. When the target enters the radar power radiation range, the radar will detect and track the target. However, the communication BS can interfere with the radar system in normal operation. In addition, the radar signals can also interfere with the communication system. Therefore, it is necessary to conduct a reasonable game analysis on the power allocation strategies of the two systems, so that both systems can operate well and avoid unnecessary interference.

**Figure 3:**
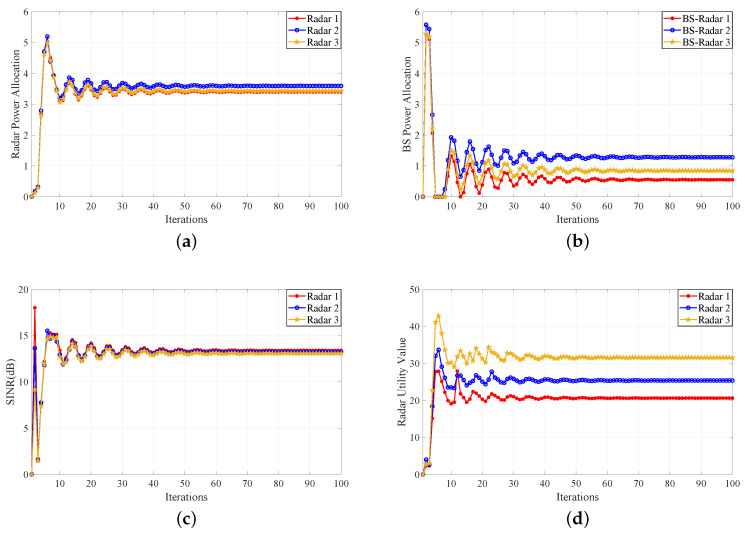
Supermodular PAG algorithm. (**a**) Power allocation convergence for multistatic MIMO radar. (**b**) Power allocation convergence for base station (BS). (**c**) Signal to interference noise ratio (SINR) convergence for multistatic MIMO radar. (**d**) Utility value convergence for multistatic MIMO radar.

Firstly, a numerical example is given to illustrate the supermodular game between the communication BS and the multistatic MIMO radars. On the one hand, there will be a tristatic MIMO radar network and a target on the radar system side. Each MIMO radar has 24 antennas. On the other hand, there will be a communication BS and three users on the communication system side. The PAG parameters are set as follows. The angles of arrival (AoA) of tristatic MIMO radars from the target are $\theta_{11}=1{}^{\circ}$, $\theta_{21}=12{}^{\circ}$, $\theta_{31}=20{}^{\circ}$, respectively. The AoAs of tristatic MIMO radars from the BS are $\theta_{R1}=-2{}^{\circ}$, $\theta_{R2}=-5{}^{\circ}$, $\theta_{R3}=-10{}^{\circ}$, respectively. The AoAs of the BS from tristatic MIMO radar are $\theta_{B1}=5{}^{\circ}$, $\theta_{B2}=13{}^{\circ}$, $\theta_{B3}=20{}^{\circ}$, respectively. The desired SINR for each radar is $\gamma_{R1}^{\min}=\gamma_{R2}^{\min}=\gamma_{R3}^{\min}=13.2\mathrm{dB}$. The whole scatter amplitudes of MIMO radars are set to 1. The maximum power $p_{Bl}^{\max}$ and $p_{R(kj)}^{\max}$ are 10. ε=[5,6,8], ω=[0.08,0.07,0.075]. Without generality, all noise power is set to 1. The tolerance of iteration difference is $10^{-8}$ or the end step of iteration is 100. When the target enters the radar power radiation range, the multistatic MIMO radar system needs to detect and identify the target. In order to track the target and guide the missile to strike the target accurately, it is necessary to suppress external interference. However, the BS, as the system equipment of the same radar party, will produce interference effect to the normal radar system. Therefore, the two systems should allocate power reasonably. Figure 3a shows the power allocation diagram of the radars. About the first 50 steps are the PAG between the radar system and the communication BS. Then, the game gradually converges to the NE after 50 steps. Figure 3b is the power allocation figure of the BS. When the BS begins to receive the radar signals, there will be a large fluctuation in its power allocation. After several games, the power allocation also converges to the NE. Figure 3c,d show the iterative variation of SINR and utility values of the radar, respectively. Similarly, the early step is the game process, and the game also converges to the NE. The best power allocation strategy for radar is to meet the accurate SINR criterion. Furthermore, the best power allocation of radar can not only achieve the expected benefits of detecting and tracking targets, but also reduce the power consumption. Meanwhile, the best power allocation of BS is to meet the QoS of MU and reduce the interference to radar. In this way, it not only ensures the stability of radar communication coexistence, but also reduces the power consumption of the two systems. Therefore, when the BS interferes with the radar, decision maker can use the supermodular PAG algorithm to allocate power between the BS and the radar reasonably. Furthermore, the algorithm can improve their work efficiency.

**Figure 4:**
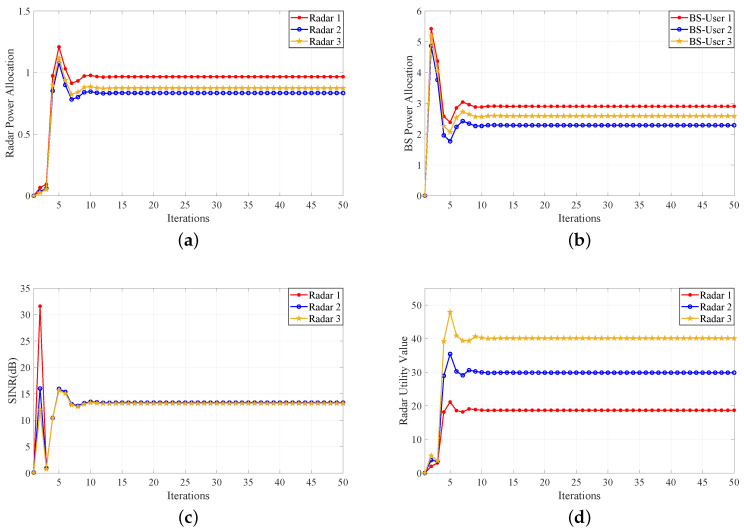
Stackelberg PAG Algorithm 1. (**a**) Power allocation convergence for multistatic MIMO radar. (**b**) Power allocation convergence for BS. (**c**) SINR convergence for multistatic MIMO radar. (**d**) Utility value convergence for multistatic MIMO radar.

**Figure 5:**
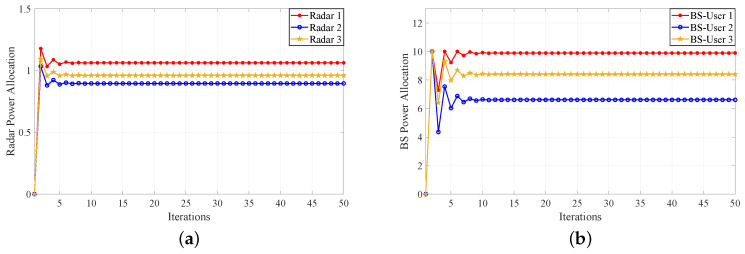

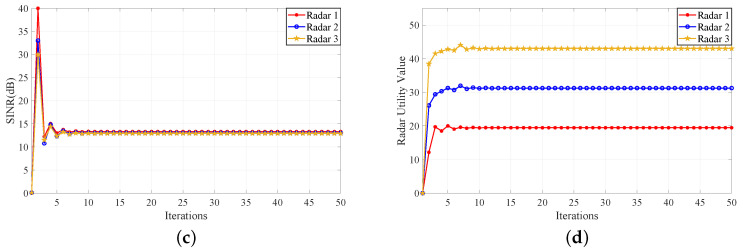
Stackelberg PAG Algorithm 2. (**a**) Power allocation convergence for multistatic MIMO radar. (**b**) Power allocation convergence for BS. (**c**) SINR convergence for multistatic MIMO radar. (**d**) Utility value convergence for multistatic MIMO radar.

Next, another numerical example shows two Stackelberg games between the multistatic MIMO radar and MU communication. Similarly, the parameters are set to the same as that of the supermodular game. For the first Stackelberg game, the radar system as leader and the BS as follower execute the power allocation. In addition, the parameters are set to μ=[5,6,8] and λ=[0.15,0.2,0.25]. The AoAs of the BS from the MU are $\theta_{C1}=-20{}^{\circ}$, $\theta_{C2}=20{}^{\circ}$, $\theta_{C3}=40{}^{\circ}$, respectively. The end step of iteration is 50. The AoAs of the radars from the MU are $\left[\theta_{R(11)},\theta_{R(12)},\theta_{R(13)}\right]=\left[45{}^{\circ},49{}^{\circ},59{}^{\circ}\right]$, $\left[\theta_{R(21)},\theta_{R(22)},\theta_{R(23)}\right]=\left[-23{}^{\circ},-26{}^{\circ},-39{}^{\circ}\right]$, $\left[\theta_{R(31)},\theta_{R(32)},\theta_{R(33)}\right]=\left[64{}^{\circ},48{}^{\circ},57{}^{\circ}\right]$, respectively. Figure 4a shows the power allocation of the radar system. The first 10 steps are the PAG of between the radar system and the communication system. Then, after the first 10 steps, the game converges to the NE quickly. Figure 4b shows the power allocation of the BS for MU. It can be seen from the diagram that the power of the BS varies greatly and converges to the NE rapidly. Figure 4c,d show the iterative variation of SINRs and utility values of the radars, respectively. It can be seen that the Stackelberg PAG converges to the NE in the end. In addition, for the second Stackelberg game, Figure 5a shows the power allocation of the radar system. About the first 10 steps are the PAG between radar system and communication system. Then, after the first 10 steps, the game converges to the NE gradually. Figure 5b is the power allocation of the BS for MU. It can be seen from the figure that the power of BS varies greatly. In the initial stage of the game, the BS costs a large amount of power, some of which reach the maximum power limit of MU. Figure 5c,d show the iterative variation of SINRs and utility values of the radars, respectively. Accordingly, if only considering the utility function of the radar system, then the supermodular PAG algorithm will only meet the utility demand for the radar side. If the utility functions of radar and user are considered at the same time, the two Stackelberg PAG algorithms can be applied to different revenue demands. In addition, the BS power allocation of the Stackelberg PAG Algorithm 2 will reach the maximum value of BS power. However, the BS power allocation of the Stackelberg PAG Algorithm 1 can be stable between the maximum and minimum power. Therefore, it can be seen from the figures that the Stackelberg PAG Algorithm 1 is more stable than the Stackelberg PAG Algorithm 2. Furthermore, the Stackelberg PAG Algorithm 1 has less radar power consumption and higher revenue than these of the supermodular PAG algorithm. In a word, the Stackelberg PAG Algorithm 1 shows better power allocation performance than that of the other algorithms.

## 6. Conclusions

This paper studies the game problem between a multistatic MIMO radar system and an MU communication system. Firstly, according to the radar jamming from the BS, a supermodular game framework is established, and the existence and uniqueness of the NE of the game are proved. With respect to the game, this paper has proposed a supermodular PAG algorithm, which converges to the NE of the game. Secondly, on the basis of the radar system jamming to communication system, two Stackelberg game theoretic frameworks are constructed. Similarly, the existence and uniqueness of NE of the two games are also proved by theoretic analysis. The corresponding PAG algorithms are proposed and converge to the NEs of the games. In addition, the supermodular PAG is mainly to satisfy the utility function of radar system, without considering the revenue of communication system. The Stackelberg PAGs consider the utility function of the two systems respectively. Therefore, the Stackelberg PAGs are more complete than the supermodular PAG. Furthermore, under the same SINR condition, the iterative convergence speeds of the Stackelberg PAG algorithms are faster than that of the supermodular PAG algorithm, and the radar power consumption is less. Meanwhile, the power consumption of radar and BS in the Stackelberg PAG algorithm 1 is less than that of the Stackelberg algorithm 2. In this way, the Stackelberg PAG algorithm 1 is a relatively superior power allocation scheme. Finally, the feasibility and convergence for the proposed algorithm are verified by numerical results.

## References

[1] Chiriyath A.R., Paul B., Bliss D.W. (2017). Radar-Communications Convergence: Coexistence, Cooperation, and Co-Design. IEEE Trans. Cogn. Commun. Netw..

[2] Li J., Stoica P. (2009). MIMO Radar Signal Processing.

[3] Yu Y., Sun S., Madan R.N., Petropulu A. (2014). Power allocation and waveform design for the compressive sensing based MIMO radar. IEEE Trans. Aerosp. Electron. Syst..

[4] Chernyak V.S. (1998). Fundamentals of Multisite Radar Systems: Multistatic Radars and Multistatic Radar Systems.

[5] Wang L., Wang L., Zeng Y., Wang M. (2017). Jamming power allocation strategy for MIMO radar based on MMSE and mutual information. IET Radar Sonar Navig..

[6] Liu Y., Liao G., Yang Z., Xu J. (2017). Multiobjective optimal waveform design for OFDM integrated radar and communication systems. Signal Process..

[7] Nowak M., Wicks M., Zhang Z., Wu Z. (2016). Co-designed radar-communication using linear frequency modulation waveform. IEEE Aerosp. Electron. Syst. Mag..

[8] Liu F., Zhou L., Masouros C., Li A., Luo W., Petropulu A. (2018). Toward Dual-functional Radar-Communication Systems: Optimal Waveform Design. IEEE Trans. Signal Process..

[9] Liu F., Masouros C., Li A., Sun H., Hanzo L. (2018). MU-MIMO Communications With MIMO Radar: From Co-Existence to Joint Transmission. IEEE Trans. Wirel. Commun..

[10] Geng Z., Xu R., Deng H., Himed B. (2018). Fusion of radar sensing and wireless communications by embedding communication signals into the radar transmit waveform. IET Radar Sonar Navig..

[11] Gu Y., Zhang L., Zhou Y., Zhang Q. (2018). Embedding Communication Symbols into Radar Waveform with Orthogonal FM Scheme. IEEE Sens. J..

[12] Hassanien A., Amin M.G., Zhang Y.D., Ahmad F. (2016). Dual-Function Radar-Communications: Information Embedding Using Sidelobe Control and Waveform Diversity. IEEE Trans. Signal Process..

[13] Blunt S.D., Yatham P., Stiles J. (2010). Intrapulse Radar-Embedded Communications. IEEE Trans. Aerosp. Electron. Syst..

[14] Gaglione D., Clemente C., Ilioudis C.V., Persico A.R., Proudler I.K., Soraghan J.J. Fractional fourier based waveform for a joint radar-communication system. Proceedings of the 2016 IEEE Radar Conference (RadarConf).

[15] Zhang J.A., Huang X., Guo Y.J., Yuan J., Heath R.W. (2019). Multibeam for Joint Communication and Radar Sensing Using Steerable Analog Antenna Arrays. IEEE Trans. Veh. Technol..

[16] Liu F., Masouros C. Hybrid Beamforming with Sub-arrayed MIMO Radar: Enabling Joint Sensing and Communication at mmWave Band. Proceedings of the 2019 IEEE International Conference on Acoustics, Speech and Signal Processing (ICASSP).

[17] Liu F., Masouros C., Li A., Ratnarajah T. (2017). Robust MIMO Beamforming for Cellular and Radar Coexistence. IEEE Wirel. Commun. Lett..

[18] Deng H., Himed B. (2013). Interference Mitigation Processing for Spectrum-Sharing Between Radar and Wireless Communications Systems. IEEE Trans. Aerosp. Electron. Syst..

[19] Singh K., Biswas S., Taghizadeh O., Ratnarajah T. Beamforming Design for Coexistence of Full-duplex Multi-cell MU-MIMO Cellular Network and MIMO Radar. Proceedings of the 2019 IEEE International Conference on Acoustics, Speech and Signal Processing (ICASSP).

[20] Singh K., Biswas S., Ratnarajah T., Khan F.A. (2018). Transceiver Design and Power Allocation for Full-Duplex MIMO Communication Systems With Spectrum Sharing Radar. IEEE Trans. Cogn. Commun. Netw..

[21] Shi C., Wang F., Salous S., Zhou J. Distributed Power Allocation for Spectral Coexisting Multistatic Radar and Communication Systems Based on Stackelberg Game. Proceedings of the 2019 IEEE International Conference on Acoustics, Speech and Signal Processing (ICASSP).

[22] Zhou Y., Zhou H., Zhou F., Wu Y., Leung V.C.M. (2019). Resource Allocation for a Wireless Powered Integrated Radar and Communication System. IEEE Wirel. Commun. Lett..

[23] Khawar A., Abdelhadi A., Clancy C. (2015). Target Detection Performance of Spectrum Sharing MIMO Radars. IEEE Sens. J..

[24] Chiriyath A.R., Paul B., Jacyna G.M., Bliss D.W. (2016). Inner Bounds on Performance of Radar and Communications Co-Existence. IEEE Trans. Signal Process..

[25] Khawar A., Abdelhadi A., Clancy T.C. (2016). Coexistence Analysis Between Radar and Cellular System in LoS Channel. IEEE Antennas Wirel. Propag. Lett..

[26] Surender S.C., Narayanan R.M., Das C.R. Performance Analysis of Communications amp; Radar Coexistence in a Covert UWB OSA System. Proceedings of the 2010 IEEE Global Telecommunications Conference GLOBECOM.

[27] Chalise B.K., Amin M.G. (2018). Performance tradeoff in a unified system of communications and passive radar: A secrecy capacity approach. Digit. Signal Process..

[28] Rihan M., Huang L. (2018). Non-orthogonal multiple access based cooperative spectrum sharing between MIMO radar and MIMO communication systems. Digit. Signal Process..

[29] Ben Kilani M., Gagnon G., Gagnon F. (2018). Multistatic Radar Placement Optimization for Cooperative Radar-Communication Systems. IEEE Commun. Lett..

[30] He Q., Wang Z., Hu J., Blum R.S. (2019). Performance Gains From Cooperative MIMO Radar and MIMO Communication Systems. IEEE Signal Process. Lett..

[31] Li B., Petropulu A.P. (2017). Joint Transmit Designs for Coexistence of MIMO Wireless Communications and Sparse Sensing Radars in Clutter. IEEE Trans. Aerosp. Electron. Syst..

[32] Alpcan T., Basar T., Srikant R., Altman E. CDMA uplink power control as a noncooperative game. Proceedings of the 40th IEEE Conference on Decision and Control (Cat. No.01CH37228).

[33] Nguyen D.H.N., Le-Ngoc T. (2011). Multiuser Downlink Beamforming in Multicell Wireless Systems: A Game Theoretical Approach. IEEE Trans. Signal Process..

[34] Moragrega A., Closas P., Ibars C. (2013). Supermodular Game for Power Control in TOA-Based Positioning. IEEE Trans. Signal Process..

[35] Bachmann D.J., Evans R.J., Moran B. (2011). Game Theoretic Analysis of Adaptive Radar Jamming. IEEE Trans. Aerosp. Electron. Syst..

[36] Deligiannis A., Lambotharan S., Chambers J.A. (2016). Game theoretic analysis for MIMO radars with multiple targets. IEEE Trans. Aerosp. Electron. Syst..

[37] Chen H., Ta S., Sun B. (2015). Cooperative Game Approach to Power Allocation for Target Tracking in Distributed MIMO Radar Sensor Networks. IEEE Sens. J..

[38] Shi C., Qiu W., Wang F., Salous S., Zhou J. (2019). Power control scheme for spectral coexisting multistatic radar and massive MIMO communication systems under uncertainties: A robust Stackelberg game model. Digit. Signal Process..

